# Therapeutic Approaches for Renal Colic in the Emergency Department: A Review Article

**DOI:** 10.5812/aapm.16222

**Published:** 2014-02-13

**Authors:** Samad EJ Golzari, Hassan Soleimanpour, Farzad Rahmani, Nahid Zamani Mehr, Saeid Safari, Yaghoub Heshmat, Hanieh Ebrahimi Bakhtavar

**Affiliations:** 1 Medical Philosophy and History Research Center, Tabriz University of Medical Sciences, Tabriz, Iran; 2 Cardiovascular Research Center, Tabriz University of Medical Sciences, Tabriz, Iran; 3 Emergency Medicine Department, Tabriz University of Medical Sciences, Tabriz, Iran; 4 Students Research Committee, Tabriz University of Medical Sciences, Tabriz, Iran; 5 Anesthesiology and Critical Care Department, Iran University of Medical Sciences, Tehran, Iran

**Keywords:** Renal Colic, Lidocaine, Nerve Block, Emergency Department

## Abstract

**Context:**

Renal colic is frequently described as the worst pain ever experienced, and management of this intense pain is necessary. The object of our review was to discuss different approaches of pain control for patients with acute renal colic in the emergency department.

**Evidence Acquisition:**

Studies that discussed the treatment of renal colic pain were included in this review. We collected articles from reputable internet databases.

**Results:**

Our study showed that some new treatment approaches, such as the use of lidocaine or nerve blocks, can be used to control the severe and persistent pain of renal colic.

**Conclusions:**

Some new approaches are discussed and their impact on renal colic pain control was compared with traditional therapies. The effectiveness of the new approaches in this review is similar or even better than in traditional treatments.

## 1. Context

Urolithiasis has been known as a very common disease for centuries (1). Renal colic presents as acute renal colic pain in the flanks due to the passage of a stone from the ureter. The classic presentation of acute renal colic is a pain radiating from the flanks to the groin and accompanied by; microscopic hematuria (85% of patients), nausea, and vomiting. Another important finding is costovertebral angle tenderness (2, 3). In some cases, urinary infection, hydronephrosis, and continuous colic attacks have been observed in urolithiasis patients (4).

Approximately 8-15% of Europeans and North Americans will experience urolithiasis (5). A total of 12% of the population suffers from urolithiasis and about two million outpatient visits in the United States are related to kidney stones. In 2000, the economic cost of renal colic and urolithiasis was about $2.1 billion (6). In 50% of people with a history of kidney stones, recurrence rates approach nearly 50% after 10 years. Kidney stone disease in men is 2-3 times more common than in women; in addition, it is more common in adults than in the elderly and it is least common in children. The disease is more common in white people, and in warm, dry climates. Decreased liquid intake and concentrated urine are two initial factors for the development of stones. Some drugs, such as; triamterene, indinavir, and acetazolamide, have been shown to be related to kidneys stones. Available oxalate in the food regimen is also categorized as another possible cause of kidney stones; although the role of the available calcium in dietary is less known and limiting the consumption of calcium in food regimens is not recommended in the long term (1). The risk of experiencing kidney stones in members of a urolithiasis patient's family is three times higher than in other individuals (7).

Renal colic pain emerges due to; obstruction of the urinary flow by a kidney stone, increased pressure on the urinary tract wall, smooth muscle spasms of the ureter, edema and inflammation near the stone, increase in peristalsis, and pressure of the proximal stone (8, 9). Increased pressure in the urinary tract following an increase of pressure in the local blood flow, and urethral smooth muscle contractions, are the main mechanisms of pain in these patients; furthermore, there is increased sensitivity to pain (10). Tension in the renal pelvis leads to the stimulation, synthesis and local release of prostaglandins, which induces diuresis and vasodilation, which in turn increases intrarenal pressure. The direct effect of prostaglandins on the ureter causes spasms in the smooth muscles of the ureteral wall (8). Permanent obstruction of the urinary tract due to renal stones leads to the release of prostaglandins in response to the existing inflammation. Within the initial hours following an obstruction, the gradient pressure between the renal glomeruli and the renal pelvis becomes equal, and consequently the glomerular filtration rate and renal blood flow decrease. If ureteric obstruction is not resolved, renal failure can occur. The best and most effective treatments for the pain of renal colic are; the spontaneous passage of a urethral stone, stone removal, placement of a stent in the ureter, and percutaneous nephrostomy. Fortunately, most patients do not suffer from complete ureter obstruction and thus they do not face the risk of renal failure (9).

Renal colic pain is often described as the worst pain the patient has ever experienced (2, 11-13). Consequently, the use of effective pain killers like; non steroidal anti-inflammatory drugs (NSAIDS), and opioids, or a combination of medications (anti-inflammatory and spasmolytic agents), play important roles in the treatment of these patients. Recently, the use of alpha-blockers (due to decreases in the transit time of urinary tract stones), nifedipine, intravenous lidocaine and nerve blocks in the paravertebral region, are known to reduce pain in renal colic (3, 5, 11).

The majority of family physicians have a lot of experience in the treatment of renal colic during their clinical practice. In recent years, great progress has been made in detection technologies of kidney stones. Nowadays, doctors can detect a renal stone to approve or reject within minutes after confirming the diagnosis. Furthermore, when the diagnosis of urolithiasis has been made, there are clear indications for a patient's referral to a urologist (1).

## 2. Evidence Acquisition

Articles used in this review were accessed from available evidence on approaches to renal colic treatment in emergency departments. The following key words were used: Therapeutic approaches; Treatment; Renal colic; and Emergency department. We searched for systematic reviews, evidence-based clinical practice guidelines, health technology assessments, and randomized controlled trials. In addition, in order to achieve a better conclusion we used the following data bases and sites:

1. Cochrane Library
2. Medline (Ovid)
3. PubMed

In this paper we only reviewed articles published from 1998 up to 2013, and our criteria for inclusion and exclusion were as follows:

Inclusion Criteria:

- Studies on renal colic treatment
- Studies performed in the adult age group

Exclusion Criteria:

- Studies published in a language other than English

### 2.1. Analysis

**Figure 1.**
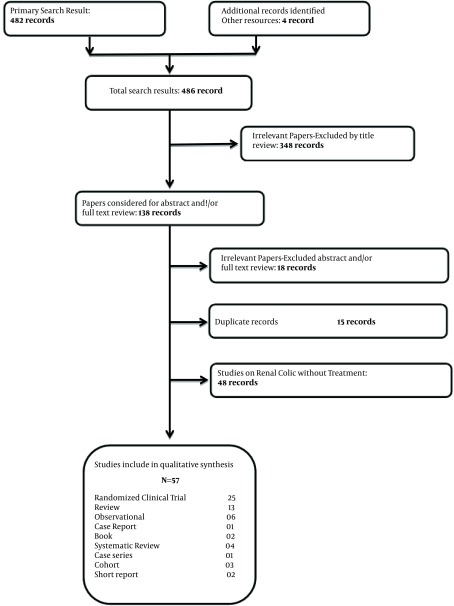
Flow Diagram of Research Papers in Our Study

The search strategy resulted in 482 articles. A title review excluded irrelevant papers (348) leaving 138 articles. Fifty seven articles were selected for further analysis including: 25 Randomized Clinical Trial, 13 review articles, 6 observational studies, 4 systematic reviews, 3 cohort studies, 2 books, 2 short reports, 1 case report, and 1 case series, which met our criteria (Figure 1). The remaining 81 articles were excluded due to the following reasons: irrelevant abstracts or full-text reviews (18 articles), duplicate records (15 articles), reviews (15 articles), and studies on renal colic without treatment (48 articles). The characteristics of all the included studies are shown in Table 1. In addition, categorizations and the number of reviewed papers are shown in Table 2. 

## 3. Results

Therapeutic approaches for the treatment of renal colic in the emergency department were introduced in the studies. However, in the studies; a common point of treatment for renal colic in the emergency department was the rapid and complete control of renal colic pain. In the following section, we investigate and explain the different types of renal colic approaches in emergency departments (Table 1). 

**Table 1.** Medications Used in the Treatment of Renal Colic

Drug	Administration Route	Study	Year
**Lidocaine**	Intravenous	Soleimanpour et al. (3, 32)	2012; 2011
		Ferrini et al. (31)	2004
**NSAIDs**	Oral	Holdgate et al. (4, 8)	2005; 2004
		Phillips et al. (6)	2009
		Davenport et al. (15)	2010
		Supervia et al. (16)	1998
		Cohen et al. (19)	1998
		Lafrance et al. (21)	2009
		Bleumink et al. (22)	2003
		Kearney et al. (23)	2006
		Grissa et al. (28)	2011
		Sakhare et al. (38)	2012
		Sumer et al. (51)	2012
	Intravenous	Safdar et al. (7)	2006
		Bektas et al. (12)	2009
		Davenport et al. (9, 15)	2005; 2010
		Cohen et al. (19)	1998
		Larkin et al. (20)	1999
		Serinken et al. (10, 24)	2008; 2012
		Morgan et al. (25)	2011
		Duggan et al. (26)	2009
		Gorocs et al. (27)	2009
		Grissa et al. (28)	2011
		Lee et al. (29)	2010
		Song et al. (36)	2012
		Kekec et al. (48)	2000
**Opioids**	Intravenous	Soleimanpour et al. (3)	2012
		Holdgate et al. (2, 4, 8)	2004; 2005
		Safdar et al. (7)	2006
		Bektas et al. (12)	2009
		Hazhir et al. (14)	2010
		Lee et al. (29)	2010
		Asgari et al. (42)	2012
		Snir et al. (43)	2008
		Yencilek et al. (44)	2008
	Intramuscular	Hazhir et al. (14)	2010
		Larkin et al. (20)	1999
		Song et al. (36)	2012
**Phloroglucinol**	Oral	Dellabella et al. (5)	2005
		Boubaker et al. (37)	2010
**Conventional and Alternative **M**ethods**	NA	Davenport et al. (9)	2005
		Nuss et al. (52)	2005
		Kober et al. (56)	2003
		Soleimanpour et al. (3, 32)	2012; 2011
**Trigger Point Injection**	NA	Iguchi et al. (11)	2002
**Hyoscine Butylbromide**	Intramuscular	Samuels et al. (33)	2009
		Kheirollahi et al. (34)	2010
		Song et al. (36)	2012
**Drotaverine Hydrochloride**		Sakhare et al. (38)	2012
		Palea et al. (39)	2006
		Romics et al. (40)	2003
**Aminophylline**	Intravenous	Barzegarnezhad et al. (45)	2012
		Nickels et al. (46)	2006
		Djaladat et al. (47)	2007
**Glycerol Trinitrate**	Topical	Hussain et al. (49)	2001
**Nifedipine **	Oral	Porpiglia et al. (50)	2000
		Dellabella et al. (5)	2005
**Alpha1-Adrenergic Blockers**	Oral	Yilmaz et al. (53)	2005
		Parsons et al. (54)	2007
		Dellabella et al. (5, 55)	2005; 2003

**Table 2.** Categorizations and Number of Reviewed Papers

Books	Original Articles	Article Reviews	Systematic Reviews	CaseControls	Case Series	Case Reports	Short Reports
**2**	31	13	4	3	1	1	2

### 3.1. Opiates

Narcotics have long been used for pain control in renal colic (5-7). Although narcotics such as morphine, codeine and meperidine for pain relief in patients with renal colic are effective, they have little effect on the underlying cause of renal colic (prostaglandins) (1). The benefits of using opioids include; low cost, good effect and titration possibility. However, the majority of physicians are not comfortable with using these drugs due to their side effects which include; nausea, vomiting, sedation, dizziness, lightheadedness, narcotic dependence, disorientation, respiratory depression, and hypotension (6, 7). Opiates can be administered in different ways, but the intravenous form is preferred because of its rapid onset. Among the different types of narcotics, morphine is used most frequently because it is stronger and less addictive than meperidine. On the other hand, there is not enough data on the effect of opiates on ureter muscle tone and there are certain conflicts; some data suggest that ureter smooth muscle tone increases with opiates, yet others suggest no effect of opiates on ureter smooth muscle tone (7-9).

Codeine and dihydrocodeine are weaker than morphine, but will relieve mild to moderate pain. Constipation is one of the main side effects of these drugs, so the long-term use of these drugs is limited. The potency of dextropropoxyphene is half that of codeine, but it can be used in combination with paracetamol (co-proxamol) for the treatment of mild pain (in contraindications of opioids). Little evidence is available on the superiority of this combination over paracetamol (9, 12).

Tramadol is another narcotic with has fewer potential side effects such as; respiratory depression, constipation, or dependence, compared to other opiates. Tramadol is as effective as morphine in reducing moderate pain after surgery, but it is less effective in more severe pain. Common side effects of this drug include; lightheadedness, nausea, dry mouth and sedation (9). Hazhir et al. evaluating the effect of intramuscular tramadol and meperidine, concluded that the effect of 100 mg tramadol is similar to the effect of 50 mg of pethidine. Nevertheless, further studies are required to confirm the therapeutic effect of tramadol in reducing pain in renal colic patients compared to previous treatments (14).

### 3.2. Non-Steroidal Anti-Inflammatory Drugs (NSAIDs)

NSAIDs alone or in combination with opioids have been used to treat renal colic pain for a long time (6). Analgesic effects of these medications are due to the inhibition of prostaglandin synthesis. As a result, NSAIDs prevent afferent arterial vasodilation and increase vascular permeability, which cause diuresis and increased pressure within the renal pelvis. NSAIDs also reduce edema, inflammation and ureter muscular hyperactivity (10).

The effect of NSAIDs on relieving pain in acute renal colic is similar to opiates. The only disadvantage of NSAIDs, in the oral or rectal form, is the delayed onset time compared with intravenous morphine. Intravenous forms of NSAIDs are available and have a rapid onset, but side effects from the intravenous form of NSAIDs have been reported more frequently than for other types of drugs. Complications of NSAIDs include; nausea, vomiting, feeling of heat or pressure in the chest, fatigue and lethargy (15).

In a meta-analysis by Holdigate et al. it was indicated that the patients for whom NDAIDs were prescribed; require less medication for pain control, experience less nausea, and have greater improvements in their pain (8).A fast-dissolving dosage form of piroxicam is similar to intramuscular sodium diclofenac in reducing pain in patients with renal colic. Moreover, due to the ease of sublingual piroxicam use, medication compliance by patients is increased (16).

In a randomized clinical trial by Phillips et al. the effect of celecoxib in the treatment of acute renal colic was studied. Patients were treated with celecoxib 400 mg, then 200 mg every 12 hours for 10 days, and then compared with a placebo-treated group. Later, it was concluded that celecoxib had no effect on the stone canal passage, but it reduced analgesic requirements (6).

During extracorporeal shock wave lithotripsy (ESWL), NSAIDs are frequently used for pain control. In their study, Labanaris et al. found that aspirin increases the risk of bleeding and prerenal hematoma (especially if accompanied by uncontrolled hypertension) during this treatment; however, no evidence of increased bleeding risk was reported with other NSAIDs (17).

Gastrointestinal side effects of NSAIDs are due to the inhibition of gastric mucosal protective prostaglandin synthesis and mucosal damage caused by stomach acid. The use of long-acting and slow release forms of NSAIDs increases the risk of upper gastrointestinal bleeding. The relative risk of upper gastrointestinal bleeding caused by NSAIDs is about 4.5 (3.8-5.3). The risk in ibuprofen (2.6),when compared with ketorolac (14.54), is obviously low,while indomethacin is 5.4 and diclofenac is 3.98 (18).

Cohen et al. in their review,' A Comparison in Effect of Diclofenac and Ketorolac in the Treatment of Renal Colic,' came to the conclusion that there is no significant difference in efficacy between the two drugs in pain relief of patients with renal colic (19). On the other hand, in a comparison with intramuscular ketorolac (60 mg) and intramuscular meperidine (150-100 mg) by Larkin et al. it was concluded that ketorolac has a better effect in reducing patients' renal colic pain than meperidine (20). In addition, when administered together, intravenous morphine and ketorolac reduce pain better than either drug does alone (7).

NSAIDs also interfere with the auto-regulatory renal blood flow system and reduce renal blood flow. This effect of NSAIDs is well-tolerated in healthy subjects without renal disease, but in patients with; previous renal disease, dehydration, cirrhosis, and recent use of nephrotoxic drugs or contrast agents, they can induce renal failure. Prostaglandins cause vasodilation in afferent glomerular arteries, and play a vital role in normal glomerular perfusion and glomerular filtration rates (GFR). As NSAIDs inhibit the synthesis of prostaglandins, they lead to a contraction of afferent arteries and a reduction in renal perfusion pressure. Nausea and vomiting are often seen in patients with renal colic which can lead to dehydration and may further contribute to renal impairment (15). Lafrance et al. in their study on the risk of renal failure due to selective and non-selective nonsteroidal anti-inflammatory drugs, came to the conclusion that the risk of acute renal failure in patients taking selective cyclophosphamide oxygenase (COX) enzyme inhibitors is lower than in non-selective agents and (COXII) enzyme inhibitors; therefore, diclofenac, ibuprofen, naproxen and ketorolac are less risky for renal failure (21).

In patients with previous heart disease, the use of NSAIDs may lead to the development of heart failure and cardiac decompensation, due to increased peripheral vascular resistance and decreased renal perfusion, in patients with impaired ventricular function and compensatory increased vasodilator prostaglandins (15). In a review of NSAIDs and heart failure, Bleumink et al. concluded that a reduction in renal blood flow, glomerular filtration, and sodium excretion, increased the load on fluids and increased systemic vascular resistance, which may contribute to the risk of kidney failure in susceptible patients (22).

The risk of coronary events increases in NSAIDs consumers. COX II inhibitors increase the cause of coronary events and myocardial infarction by 25%. High-dose ibuprofen (800 mg 3 times daily) and high-dose diclofenac (75 mg 2 times daily) have also been associated with the risk of coronary events; however, naproxen (500 mg 2 times a day) risk in causing a coronary event is lower. It is assumed that this effect is due to an inhibition of platelet aggregation (23).

### 3.3. Alternative Treatments

#### 3.3.1. Paracetamol

Paracetamol (acetaminophen) is a safe and effective analgesic with fewer side effects than NSAIDs and opiates. The drug can be administered orally, rectally or intravenously (12). Despite 50 years of research conducted on acetaminophen, the actionmechanism of this drug has still not been fully characterized (24). However, due to significant concentrations in the cerebrospinal fluid after administration of the drug, it is believed that this drug works on the central nervous system (25). Acetaminophen acts by inhibiting prostaglandin synthesis (which are free of inflammatory response) (26), furthermore, metabolites of acetaminophen with N-arachidonoylaminophenol (AM404) inhibit endogenous cannabinoids, such as an andamide reuptake in the synaptic cleft, and consequently they cause analgesic effects (24). Intravenous, compared with oral or rectal acetaminophen, has a more rapid onset time thanks to its direct entry into the bloodstream (27). Acetaminophen is well-tolerated and side effects are rare, but it can cause; weakness, hypotension and elevated liver enzymes (26). In patients with renal failure because of delayed drug elimination, the drug half-life increases to five hours; the recommended interval between the administration of the drug in these patients is six hours (25). Bektas et al. in their study comparing the effects of paracetamol and morphine on renal colic pain relief concluded that the effect of intravenous acetaminophen in pain reduction is much better and it has fewer side effects than intravenous morphine (12). Grrisa et al. in another study comparing the effects on renal colic in pain relief between paracetamol and piroxicam, suggested that pain relief following the administration of a single dose of intravenous paracetamol was superior to intramuscular piroxicam (28). Lee et al. in their study on the comparative effects of paracetamol and morphine for the treatment of pain after thyroidectomy, concluded that the effect of 1g of intravenous paracetamol is similar to 30 mg of ketorolac on pain relief, so when NSAIDs are contraindicated, intravenous paracetamol could be an option (29).

#### 3.3.2. Lidocaine

Lidocaine has become the agent of choice in visceral and central pain. On the other hand, when narcotics are not effective, the administration of lidocaine is a useful alternative and there are no unwanted side effects similar to that of other narcotics. Intravenous lidocaine is effective in the management of neuropathic pain such as; diabetic neuropathy, post-surgical pain, post herpetic pain, headaches, and neurological malignancies (3). Lidocaine is an anesthetic amide that reversibly blocks voltage-dependent sodium channels and thus it may lead to the inhibition of nerve impulse transmissions. Systemic toxicity associated with the administration of lidocaine occurs because it blocks sodium channels in the heart and brain. Poisoning symptoms can range from; mild neurologic symptoms, to intractable seizures, and cardiovascular collapse (30). However, if administered at low doses, lidocaine is a relatively safe medication. Allergy to lidocaine leads to an increased risk of cardiac dysrhythmias and dyspnea in some patients. Lidocaine is effective, inexpensive and has few side effects including; lightheadedness, nausea and constipation. Overall, the incidence of side effects compared to those of other drugs and narcotic analgesics is low. On the other hand, a wide range of adverse effects of lidocaine are predictable, but due to the lower half-life of lidocaine, lidocaine toxicity symptoms are transient and rapidly reversible (3).

Ferrini et al. in their review on the use of lidocaine in the treatment of severe pain or neuropathic pain, concluded that lidocaine is more effective in reducing visceral and central pain compared to narcotics. Furthermore, when the use of narcotics is not effective or side effects are observed in patients, lidocaine is appropriate (31-32). Soleimanpour et al. in their study compared the effects of intravenous lidocaine and morphine in the treatment of renal colic in the emergency department and found that lidocaine significantly reduces pain compared to morphine in patients with renal colic (3). 

#### 3.3.3. Hyoscine Butyl Bromide

Anti-muscarinic agents are effective in the treatment of smooth muscle spasms (especially gastrointestinal). Ureteral peristaltic activity of the genitourinary system is controlled by the autonomic nervous system so the use of anti-muscarinic agents can be effective (9). Hyoscine butyl bromide (Buscopan TM) is an anti-muscarinic drug which blocks the action of acetylcholine at the parasympathetic nerve endings in muscles and glands (33), and theoretically it is effective when administered in relieving pain associated with analgesic drugs for moderate renal colic pain (2).

Holdgate et al. in their review on the role of anti-muscarinic agents (hyoscine butyl bromide) in renal colic, reported that Buscopan has no effect in reducing renal colic pain and it does not reduce the need for additional opiates (2). In their study, Kheirollahi et al. compared the effect of combined intranasal desmopressin and intramuscular hyoscine with hyoscyamine alone in acute renal colic, and they found that the combination of hyoscine with desmopress in alone is more effective in renal colic (34). Kheirollahi et al., in their study comparing the effect of hyoscine with diclofenac and diclofenac alone in the treatment of renal colic, concluded that the combination of diclofenac and hyoscine compared with diclofenac alone, resulted in rapid improvement of renal colic pain (34). Song et al. in their review on the effect of adding hyoscine to ketorolac and morphine in patients with renal colic, stated that the combination of morphine and hyoscine with ketorolac creates a greater reduction in patients' pain. This decrease is statistically significant, but clinically the reduction in pain intensity is not significant (36).

Hyoscine butyl bromide has obvious side effects including: dry mucous membranes, photophobia, facial flushing, dry skin, loss of accommodation, constipation, urinary retention, and renal colic thus limiting its routine administration (9, 35-36).

#### 3.3.4. Phloroglucinol

Phloroglucinol is a drug with potent antimuscarinic effects that are well-tolerated (37), although little evidence about the effectiveness of these drugs is available for renal colic. In their study, Boubaker et al. evaluated the analgesic effect of this drug in combination with piroxicam on renal colic, it was suggested that phloroglucinol has no effect in reducing the severity of pain when added to piroxicam (37).

#### 3.3.5. Drotaverine

Drotaverine is an inhibitor of Phosphodiesterase 4 (PDE4) in smooth muscles and it has anti-spasmodic activity without anti-muscarinic adverse effects. This drug is structurally similar to papaverine and its structural formula is Diethoxy-1, 2, 3, 4-tetrahydroisoquinoline 1 - (3, 4-diethoxybenzylidene) -6, 7 (38). Drotaverine provides effective analgesia and is used in the treatment of renal colic (39). Palea et al. in their review on the effects of drotaverine relaxation on the human urethral muscle ring, found that the effect of drotaverine relaxation on the muscles of the humanureter is similar to that of other Animal and its power is approximately six times stronger than papaverine: hence, the ureteral muscle relaxant advantage can be used in the treatment of renal colic patients (39). Romics et al. in their study on the impact of drotaverine on renal colic pain, reported that pain intensity was reduced in more than two-thirds of patients with intravenous drotaverine. Drotaverine’s side effects are not significant, but include; transient drop in blood pressure, dizziness, nausea and vomiting (40).

#### 3.3.6. Papaverine Hydrochloride

Papaverine, 1 - [(3, 4-dimethoxyphenyl) methyl] -6,-7-dimethoxyisoquinoline, results in smooth muscle relaxation and is found in opiate compounds. Due to the relaxation properties of this drug, it is used in an intra-arterial injection, as a vasodilator in cerebral vasospasm after a subarachnoid hemorrhage. Furthermore, it is also used as a therapeutic agent in erectile dysfunction (impotence) and renal colic. Its pharmacological effects are due to blocking of the voltage-dependent potassium channels (41).

Asgari et al. in their review on the effects of papaverine in the treatment of renal colic, concluded that adding papaverine to diclofenac, compared with diclofenac alone, has a greater impact in reducing pain. The only reported side effect of papaverine is dizziness, which occurs in only a small percentage of patients (2%) (42). Snir et al. concluded that using papaverine in the treatment of renal colic is as effective as sodium diclofenac in the management of patients with renal colic pain in the short term, and can be effective if there are contraindications for the use of NSAIDs; however, the analgesic effect of diclofenac in controlling pain lasts longer than papaverine (43). Yencilek et al. reported that papaverine is more effective in renal colic pain relief in patients with refractory pain than conventional agents (44).

#### 3.3.7. Aminophylline

Aminophylline is a methylxanthine drug and a derivative of theophylline. The strength and duration of aminophylline’s action is less than theophylline. It relaxes smooth muscles, especially; muscles of the bronchial walls, heart and central nervous system stimulation, and diuresis. This drug also crosses the placental barrier. Its mechanism is through the inhibition of a non-selective phosphodiesterase inhibitor that; increases the concentration of intracellular cAMP, activates PKA (protein kinase A), inhibits TNF-α and the synthesis of leukotrienes, and reduces inflammation. Non-selective antagonism of adenosine receptors is the second mechanism (45). Aminophylline is a well-known drug and it is widely used in the treatment of renal colic (46).

Djaladat et al. in their study on the effect of aminophylline on renal colic, concluded that aminophylline decreases renal colic pain in patients and reduces the need for a narcotic prescription (47). In their study using a local injection of aminophylline during trans-urethral lithotripsy, Baregarnezhad et al. showed that aminophylline facilitates the uretroscopy procedure, increases the success rate for the treatment of renal colic by using (transurethral lithotripsy), decreases operative time, and reduces the need for a double J catheter and wave lithotripsy (45).

#### 3.3.8. Nitrates

Nitrates, thanks to their impact on vascular smooth muscle, have been studied in the treatment of renal colic. Two surveys have been conducted on the effects of isosorbide dinitrate and glycerol trinitrate (9). Nitrates in vascular smooth muscle lead to the release of nitric oxide and ultimately to increased concentrations of cGMP, which interferes with guanylyl cyclase and the relaxation of smooth muscles. This effect is caused by nitrates in the genital tract smooth muscle. However, due to the short effectiveness time of nitrates, the clinical value of smooth muscle relaxant effects in the genitourinary system is low (48). Little evidence is available on the effect of these drugs on renal colic.

Kekec et al. studied the effect of adding NSAIDs to isosorbide dinitrate in the treatment of renal colic and came to the conclusion that adding isosorbide to tenoxicam, compared with tenoxicam alone, results in a decrease in pain severity, and this reduction in pain intensity is clinically significant. Side effects observed in this study were; headaches, flushing and orthostatic hypotension (48).

Hussain et al. in their study on the effects of glycerol trinitrate patches (GTN) in patients with ureteral stones with a diameter less than 10 mm over a period of six weeks, concluded that patients who used the GTN patches experienced less pain episodes compared with placebo groups; however, the rate was not statistically significant. During the treatment period, 27% of the patients discontinued their treatment with GTN because of headaches (49).

#### 3.3.9. Calcium Channel Blockers

In-vitro studies have shown that calcium channel blockers decrease ureteral human peristalsis. Calcium is essential for the maintenance of action potential in the ureter ductand also for contraction of the ureter. Calcium channel blockers inhibit the influx of calcium, and therefore are expected to have an inhibitory effect on the activity of the ureter. Nifedipine appears to be the most effective drug in this category (5, 9). Little evidence for the effectiveness of nifedipine in acute renal colic or its role in facilitating the passage of duct stones is available.

In a study by Porpiglia et al. (50), it was found that although nifedipine does not reduce pain in the acute phase, it can lead to stone removal from the channel in a short time and decrease analgesic requirements. Due to their anti-inflammatory effects, steroids are administered with nifedipine; although they are effective, the risks of long-term steroids use should be considered.

#### 3.3.10. Alpha-Blocker

Ureteral activity is regulated by the autonomic nervous system. In the sympathetic nervous system, α-fibers are stimulants and β-adrenergic inhibitory (9), in addition, α-1 adrenergic receptors are available in the human ureter. These receptors, especially subgroup 1Dα, play roles in the dilatation of detrusor muscles and spasms of the distal third of the ureter (51). Blocking alpha-adrenergic receptors reduces spasms and pain, and in addition it induces ureteral stone removal (52).

Yilmaz et al. treated renal colic in patients who had distal ureteral stones with alpha-adrenergic blockers, and they found that the use of these drugs facilitates the removal of ureteral stones (53). Parsons et al. in their study on patients with ureteral stones, showed that alpha-blockers may lead to increased rates of urethral stones removal (54). Sumer et al. divided patients with urolithiasis into three groups, and treated each group separately with; diclofenac and alpha-blocker combination, diclofenac and prednisolone combination, and the third group received diclofenac alone. In the group treated with adiclofenac and alpha-blocker combination, stone removal rates were higher in comparison with the other two groups. Alpha-blocker drugs are used more often in combination with antibiotics and oral corticosteroid therapy for medical expulsive therapy (MET) (51).

MET is considered in the treatment of patients with uncomplicated distal ureteral stones before uretroscopy or extracorporeal lithotripsy. This method increases the success rate of stone removal and decreases ureteral colic. In newly-diagnosed patients with ureteral stones less than 10 mm in diameter, the symptoms are controlled; however, periodic assessment and observation of patients is necessary. In these patients, the use of MET is considered in order to facilitate the removal of duct stones. There is evidence that MET reduces the need for the administration of analgesia and accelerates the passage of ureteral stones with diameters less than 10 mm (similar size pieces of stone after shock wave lithotripsy (SWL)). Evidences suggest that MET could be considered as an effective treatment (51).

Dellabella et al. have suggested that prescribing tamsolosin results in stone removal in almost all patients in a very short time, without the need for hospitalization. In another research on the effects of tamsolosin on drug treatment of juxtavesical ureteral stones, it was concluded that the use of tamsolosin in patients increases stone removal rates, decreases the need for hospitalization and endoscopic procedures, as well as providing better control of the patients' colic pain (55).

#### 3.3.11. Trigger Point Injection

Trigger point injection (TPI) has been used to control pain in patients with chronic, visceral, and myofascicular pain. In patients with renal colic, a local anesthetic is injected into the ipsilateral posterior surface of the trigger points. In this method, the patient is placed in a prone position andgentle pressure is used over the trigger points with the end of a ballpoint pen, and then markedat intervals of 1 cm in the triangular area bounded by the edges of the ribs, spine and iliac crest. When pressure is applied and the patient feels pain, the trigger points are determined. Subsequently, 2-3 mL of lidocaine 1% is injected into an area of about 3 cm in diameter, and using a long (23 gauge, 6 cm) needle, 5-10 mL of 1% of lidocaine is injected into the deep portion of the psoas muscle (3, 11). Iguchi et al. used this method for pain control in patients with renal colic, and concluded that the combination of sulpyrine and butyl scopolamine was more effective and resulted in faster pain control. This method is also simple, effective and safe (11). Other alternative techniques such as subcutaneous paravertebral nerve block, and sympathetic chain block and catheter insertion into the upper lumbar sympathetic chain for continuous infusion, have also been used successfully in the treatment of renal colic (3, 11).

### 3.4. Non-Medicated Alternative Therapies

#### 3.4.1. Local Active Warming

Local heating has been suggested as a way to reduce pain in trauma patients in the emergency department. Localized heating of the active region of the abdomen and lower back can be a simple and effective method for pain control in patients with suspected renal colic during patient transportation to the emergency department and hospital. Kober et al. reported that local heating in the abdominal and back areas of patients with renal colic, significantly decreases anxiety and pain (56).

#### 3.4.2. Acupuncture

Acupuncture has long been used in China and Taiwan, and its analgesic effects appear faster compared to conventional analgesic therapies, furthermore, they are without any complications. In a study by Lee et al. concerning the effects of acupuncture on pain management for renal colic, it was determined that the effect of acupuncture compared to avofortan was quicker; hence, acupuncture was introduced as an alternative method for renal colic pain control. Acupuncture plays its role by increasing endogenous opiate levels in the cerebrospinal fluid (9).

## 4. Conclusions

Nowadays, opiates and NSAIDs are used in most countries for the control of renal colic pain. Knowing that these drugs have side effects, the administration of alternative therapies would appear to be inevitable. In the present review we presented almost all possible treatments for renal colic. However, more studies are needed to be conducted on the use of alternative therapies in renal colic. Some medications such as lidocaine and papaverine have worked well in patients resistant to conventional therapies; however, as inadequate evidence is available in this regard, further studies are needed. Furthermore, the effectiveness of rapid pain control of renal colic and complications resulting from these medications need to be reviewed in future trials.
